# Visual Responses in Mice Lacking Critical Components of All Known Retinal Phototransduction Cascades

**DOI:** 10.1371/journal.pone.0015063

**Published:** 2010-11-29

**Authors:** Annette E. Allen, Morven A. Cameron, Timothy M. Brown, Anthony A. Vugler, Robert J. Lucas

**Affiliations:** 1 Faculty of Life Sciences, University of Manchester, Manchester, United Kingdom; 2 Department of Ocular Biology and Therapeutics, University College London Institute of Ophthalmology, London, United Kingdom; Pennsylvania State University, United States of America

## Abstract

The mammalian visual system relies upon light detection by outer-retinal rod/cone photoreceptors and melanopsin-expressing retinal ganglion cells. *Gnat1^−/−^;Cnga3^−/−^*;*Opn4^−/−^* mice lack critical elements of each of these photoreceptive mechanisms via targeted disruption of genes encoding rod α transducin (*Gnat1*); the cone-specific α3 cyclic nucleotide gated channel subunit (*Cnga3*); and melanopsin (*Opn4*). Although assumed blind, we show here that these mice retain sufficiently widespread retinal photoreception to drive a reproducible flash electroretinogram (ERG). The threshold sensitivity of this ERG is similar to that of cone-based responses, however it is lost under light adapted conditions. Its spectral efficiency is consistent with that of rod opsin, but not cone opsins or melanopsin, indicating that it originates with light absorption by the rod pigment. The TKO light response survives intravitreal injection of U73122 (a phospholipase C antagonist), but is inhibited by a missense mutation of cone α transducin (*Gnat2^cpfl3^*), suggesting Gnat2-dependence. Visual responses in TKO mice extend beyond the retina to encompass the lateral margins of the lateral geniculate nucleus and components of the visual cortex. Our data thus suggest that a Gnat1-independent phototransduction mechanism downstream of rod opsin can support relatively widespread responses in the mammalian visual system. This anomalous rod opsin-based vision should be considered in experiments relying upon *Gnat1* knockout to silence rod phototransduction.

## Introduction

A well characterised signal transduction cascade links light absorption by rod and cone opsins to photoreceptor hyperpolarisation[1]. The α subunit of g-protein transducin is central to this cascade. Liberated from inhibitory β and γ subunits by photoactivated opsin, it in turn activates a cGMP phosphodiesterase to degrade cytosolic cGMP, leading to closure of cyclic nucleotide gated channels in the plasma membrane.

Several phototransduction cascade components are expressed uniquely in either rods or cones, allowing selective ablation of photoreception in either class to be achieved by genetic manipulation. Some years ago, two such transgenic mice (*Gnat1^−/−^* which lacks Gnat1, the rod specific α transducin[2]; and *Cnga3^−/−^*, lacking a cone photoreceptor-specific cyclic nucleotide channel subunit[3]), were crossed with a third lacking the melanopsin (Opn4) gene (responsible for inner retinal phototransduction [4]). As each of these knockouts had been shown previously to abolish phototransduction in the target photoreceptor class, it was assumed that combining them to produce *Gnat1^−/−^;Cnga3^−/−^;Opn4^−/−^* (herinafter TKO) mice would result in mice lacking functional photoreception[5]. These mice did indeed lack several visual behaviours that are retained in the absence of rods and cones (circadian photoentrainment and photic suppression of wheel running activity), leading to the conclusion that together rods, cones and melanopsin account for all light detection in the mammalian retina[5].

That view has received independent confirmation[6]. Nonetheless, the original publication with these mice reported that they did not entirely lack photosensitivity as they retained a residual pupillary light reflex at high irradiances. The magnitude of the pupillomotor response in these TKO mice was very small and its appearance unreliable both between and within individuals. As a result we were unable to explain this surprising finding, except to suggest that it might originate with the photoreceptor early receptor potential - a light-dependent charge displacement, which is not reliant upon the phototransduction cascade[7].

In an unrelated experiment we recently included TKO mice as a negative control in some electroretinogram (ERG) recordings. We were surprised to discover that these animals retain a reproducible flash ERG. Here we provide a full characterisation of this ERG, and show that visual responses in the TKO mice can be recorded also in a sub-compartment of the lateral geniculate nucleus and the visual cortex. Our data suggest that a rod opsin-based Gnat1-independent phototransduction mechanism can support widespread visual responses in mice.

## Materials and Methods

Mice were bred and housed at University of Manchester or the Institute of Ophthalmology under a 12∶12 light dark cycle, with food and water available *ad libitum*. Both male and female mice were used, aged 3-6 months. All procedures conformed to requirements of the UK Animals (Scientific Procedures) Act, 1986, and were approved by the Home Office under project licence number 40/2911. Light measurements employed calibrated spectroradiometers (Bentham Instruments, Reading, UK; Ocean Optics, FL, USA) and an optical power meter (Macam Photometrics, Livingston, UK). Where appropriate the scotopic luminance (cd/m2) of stimuli was calculated by correcting spectral irradiance profiles (in W/m2/nm) according to the sensitivity of mouse rods (as approximated by a λ_max_ = 498 nm nomogram [8]), and renormalising by photon energy, before summing across wavelengths and multiplying by 1700.

### Electroretinography

Mice were long-term dark adapted (>12 hr) and prepared for electroretinography under dim red light (<−0.6 log_10_ cd/m^2^ >650 nm). Anaesthesia was induced by intraperitoneal ketamine (70 mg/kg) and xylazine (7 mg/kg) and maintained using subcutaneous top-up injections of (72 mg/ml) and xylazine (5 mg/ml). A topical mydriatic (tropicamide, 1%, and phenylephrine, 2.5%; Chauvin Pharmaceuticals, UK) and hydroxypropyl methylcellulose solution (0.5%; Alcon Laboratories, Ltd., UK) were applied to the recording eye prior to placement of a corneal contact-lens type electrode. A silver wire bite bar provided head support and acted as a ground, and a needle reference electrode (Ambu® Neuroline) was inserted approximately 5 mm from the base of contralateral eye. Electrodes were connected to a Windows PC via a signal conditioner (Model 1902 Mark III, CED, UK), which differentially amplified (×3000) and filtered (band-pass filter cut-off 0.5 to 200 Hz) the signal, and a digitizer (Model 1401, CED). Core body temperature was maintained at ∼37°C throughout recording.

Dark adapted (scotopic) ERGs were elicited by 15 ms full field flashes produced by a xenon arc source (Cairn Research Ltd., UK) fitted with neutral density and interference filters as necessary to achieve corneal irradiances in the range −4.5–3.5 log_10_ scotopic cd/m^2^ (−4.8 to 3.2 log_10_ μW/cm^2^). Average response waveforms for each individual were produced from between 40 and 6 stimulus repeats applied at inter-stimulus intervals ranging from 1500 ms at dimmest intensities to 30s at brightest intensities. Light adapted ERGs were elicited by bright white flashes (Grass Model PS33 Photic Stimulator, Astro-Med, Inc., Rhode Island, USA, fitted with a 400 nm high pass filter; 10 µs duration; peak corneal irradiance 3.6 log_10_ cd/m^2^) at a frequency of 0.75 Hz, presented against a rod-saturating background white light (metal halide source; 3.1 log_10_cd/m^2^). Data shown here are from recordings taken at least 20 min after the background light was switched on to allow for cone light adaptation. In all cases a-wave amplitude was calculated relative to baseline prior to stimulus onset, and b-wave with reference to the a-wave trough.

For analysis of spectral sensitivity the predicted spectral response functions of opsin:vitamin A based photopigments [8] with λ_max_ = 480 nm, 498 nm or 508 nm were used to approximate the spectral efficiency of mouse melanopsin, rod opsin and MWS-cone opsin respectively. These were then used as the basis for wavelength dependent corrections in stimulus irradiance.

Pharmacological agents (from Tocris Bioscience, UK) were applied by intravitreal injection. Following application of a topical analgesic (Amethocaine Hydrochloride 1%: Chauvin Pharmaceuticals, UK) a glass needle (tip approximately 20 µm) was inserted behind the limbus, and 1 µl injected. Post-treatment ERG recordings were made 30 mins after injection and compared against pre-injection responses from the same eye. YM-298198 (6-Amino-N-cyclohexyl-N,3-dimethylthiazolo[3,2-a]benzimid azole-2-carboxamide hydrochloride) and Fenobam (1-(3-chlorophenyl)-3-(3-methyl-5-oxo-4H-imidazol-2-yl)urea) were prepared in distilled water, while U-73122 was first dissolved in DMSO. Injections of DMSO at the highest concentration (20%) had no effect on ERG amplitude (n = 6; data not shown).

### In Vivo Neurophysiology

Adult male mice (80–160 days) were anaesthetized by i.p. injection of 30% (w/v) urethane (1.7 g/kg; Sigma, UK) and placed in a stereotaxic apparatus (SR-15 M; Narishige International Ltd., UK). Additional top up doses of anaesthetic (0.2 g/kg) were applied as required and body temperature maintained at 37°C with a homeothermic blanket (Harvard Apparatus, Kent, UK). The skull surface was exposed and a small hole (∼1 mm diam.) drilled 2.5 mm posterior and 2.3 mm lateral to the bregma. The pupil, contralateral to the craniotomy, was dilated with topical midriatic (1% (w/v) atropine sulphate; Sigma) and the cornea kept moist with mineral oil. A recording probe (A4X8-5 mm-50-200-413; Neuronexus, MI, USA) consisting of 4 shanks (spaced 200 µm), each with 8 recordings sites (spaced 50 µm) was then positioned centrally on the exposed skull surface, perpendicular to the midline, and lowered to a depth of 2.2 to 3.4 mm using a fluid filled micromanipulator (MO-10; Narishige).

Once the recording probe was in position mice were dark adapted for 1 h, which also allowed neuronal activity to stabilise after probe insertion/repositioning. Neural signals were acquired using a Recorder64 system (Plexon, TX, USA). Signals were amplified x3000, highpass filtered at 300 Hz and digitized at 40 kHz. Multiunit activity (spikes with amplitudes >50 µV) were saved as time-stamped waveforms and analyzed offline (see below). Light stimuli (460 nm; half peak width: ±10 nm), were generated by a custom built LED based light source (Cairn Research Ltd.), passed through neutral density filters as necessary, and was focused onto a 5 mm diameter opal diffusing glass (Edmund Optics Inc., UK) positioned 3 mm from the eye contralateral to the recording probe. Unattenuated intensity of the 460 nm stimuli was 6.8×10^15^ photons/cm^2^/s (corresponding to 3.4×10^4^ mouse scotopic cd/m^2^). Intensity response relationships were calculated by applying 2s, 460 nm steps (interstimulus interval of 18s) spanning a 5 log unit range. At each intensity, starting at the lowest (6.8×10^11^ photons/cm^2^/s) 20 trials were completed before increasing the light intensity by a factor of 10. After recording a set of responses at one location the recording probe was raised or lowered by 400 µm and, after 1 h dark adaptation, another set of responses recorded. In some experiments this procedure was repeated another 1 or 2 times such that in individual animals our recordings spanned 16×4 to 32×4 grids in and around the LGN.

At the end of the experiment the mouse was perfused transcardially with 0.1 M phosphate buffered saline (PBS) followed by 4% paraformaldehyde. The brain was removed, postfixed overnight, cryoproteced with 30% sucrose then sectioned at 100 µm on a freezing sledge microtome. Sections were mounted with Vectashield (Vectorlaboratories Ltd. Peterborough, UK), coverslipped and the probe site reconstructed by locating a fluorescent dye (Cell Tracker CM-DiI; Invitrogen Ltd. Paisley, UK) applied to the probe prior to recording. Sections were scaled to account for shrinkage using the distance between electrode tracks and aligned with the corresponding mouse atlas sections [9] to estimate the stereotaxic coordinates of each recording site.

Multichannel, multiunit recordings were analysed in Offline Sorter (Plexon). After removing artefacts we used principal component based sorting to discriminate single units, identifiable as a distinct cluster of spikes in principal component space with a clear refractory period in their interspike interval distribution. In most cases there was a single distinct unit at each recording site (total 321 units from 512 recording sites). Following spike sorting data were exported to Neuroexplorer (Nex technologies, MA, USA) and MATLAB R2007a (The Mathworks Inc., MA, USA) for construction of peristimulus histograms and further analysis. Light responsive units were identified as those where the peristimulus average showed a clear peak (or trough) that exceeded the 99% confidence limits estimated from a Poisson distribution derived from the prestimulus spike counts.

### c-fos immunohistochemistry

TKO mice (n = 6) were dark-adapted overnight and killed the following morning by cardiac perfusion with PBS followed by 4% paraformaldehyde either under dim red light (<10 lux; n = 3), or following 90 minutes exposure to bright white light of ∼1300 lux (n = 3). Brains were post-fixed overnight at 4°C prior to cryoprotection in 30% sucrose and rapid freezing in OCT embedding matrix (Raymond A Lamb Ltd.). Coronal brain sections (30 µm) were taken through the visual cortex and processed free-floating for multiple immunolabelling as follows: Brain sections were blocked with 5% normal donkey serum (Jackson ImmunoResearch) and labelled concurrently with rabbit anti-c-fos (Calbiochem, at 1∶5000 dilution) and a mouse monoclonal antibody to non-phosphorylated neurofilament protein (SMI-32, Covance at 1∶5000). The latter has been used previously to define the cytoarchitecture of mouse visual cortex [10]. Primary antibodies were applied overnight with constant agitation, followed by incubation in secondary FITC anti-rabbit and TRITC anti-mouse antibodies (Jackson ImmunoResearch) for 2 h. All blocking and antibody incubations were performed with 0.1M PBS containing 0.3% triton X-100 (BDH) and sections were washed extensively in PBS between application of primary and secondary antibodies. Brain sections were washed in PBS, mounted onto glass slides and cover slipped using Vectashield (Vector labs).

### Retinal Immunohistochemistry

Eyes from n = 3 WT and TKO mice were fixed in 4% paraformaldehyde and 12 µm sections collected. Sections were incubated with antibodies against the α (anti-Gαt2 antibody sc-390 from Santa Cruz, diluted 1∶200 to give 1 µg/ml) or γ (anti-Gγc antibody; PAB8-00801-G from Cytosignal, Irvine, CA, diluted 1∶500) subunits of cone transducin and/or rod opsin (4D2, kind gift from Robert Molday, diluted 1∶50). Where appropriate sections were counterstained with DAPI and mounted with VectaShield. Images were acquired using a Zeiss 510 confocal microscope with LSM software. Specificity of the anti-Gαt2 antibody for it's target peptide sequence was confirmed by preadsorption with the immunising peptide (blocking peptide sc-390P from Santa Cruz) at 1∶5 dilution for 2 h at room temperature. Cross reactivity between the anti-Gγc antibody and the γ subunit of rod transducin has been specifically excluded by the manufacturers (cytosignal, Irvine CA).

## Results

### Rod-based ERG in TKO mice

**Figure 1:**
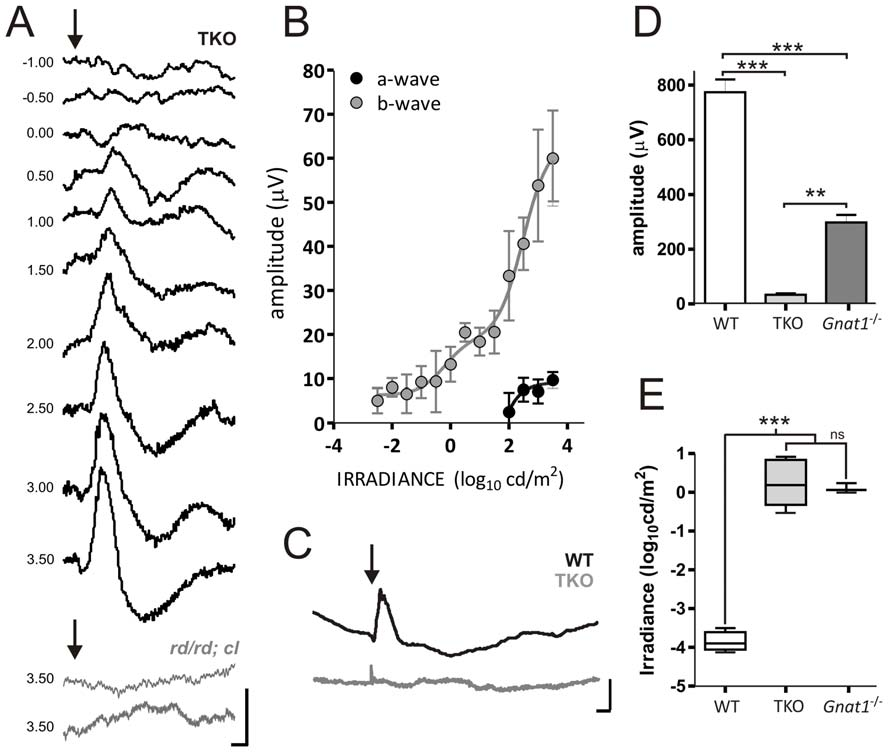
ERG responses in *Gnat1^−/−^;Cnga3^−/−^;Opn4^−/−^* (TKO) mice. **A**, Dark adapted flash ERG traces from a representative TKO mouse and representative traces from two *rd/rd cl* mice; arrow depicts flash onset; scale bar  = 50 ms (x-axis), 25 µV (y-axis); numbers to left are stimulus irradiance in log cd/m^2^. **B**, Mean (±SEM; n = 5) a- and b-wave amplitudes for flash ERG in TKO mice. **C**, Representative light-adapted ERG traces in wild type (WT) and TKO mice (Scale bar =   = 50 ms (x-axis), 25 µV (y-axis)). **D**, b-wave amplitude (mean±SEM) at the brightest flash (3.5 log_10_ cd/m^2^) in wild-type (n = 6), TKO (n = 4) *Gnat1^−/−^* mice (n = 5) compared by one-way ANOVA (p<0.001) and Bonferroni's post test. **E**, Estimated threshold irradiance (box shows median±upper lower quartiles, whiskers range of data) for a reliable ERG response in TKO (n = 5), *Gnat1^−/−^* (n = 3) and wild type mice (n = 6) compared with one-way ANOVA (p<0.0001) and bonferroni post test. *** p<0.001; ** p<0.01; ns p>0.05.

TKO mice and wild type controls were dark-adapted for >12 hrs and exposed to white light flashes of increasing irradiance (−4.5 to 3.5 log_10_ cd/m^2^). Consistent with previous reports that *Gnat1^−/−^* mice lack high sensitivity ‘scotopic’ vision[2], [11], we did not observe any response in TKO mice at low flash intensities (Fig. 1A & B). However, at irradiances >0.5 log_10_ cd/m^2^, a small positive deflection (resembling the conventional b-wave) became apparent. The amplitude and latency (time to peak, or implicit time) of this deflection was dependent on flash intensity. Even at the highest intensity, the deflection was relatively small (5–10% that of the saturating wild type response; Fig. 1D), its latency was also somewhat greater (although well within the range expected of ERG b-waves). At the higher flash intensities, a small negative deflection (corresponding to the wild type a-wave) could generally be detected preceding the major positive deflection (b-wave). This ERG response likely originates in the outer retina, as we failed to record any such response from *rd/rd cl* mice (a model of advanced rod/cone degeneration[12]) under identical conditions (Fig. 1A).

The threshold irradiance for a measurable TKO ERG (defined as the irradiance at which b-wave peak >2 standard deviations above mean baseline value for each individual) was around 1 cd/m^2^, some 3–4 decimal orders greater than that of wild types (Fig. 1E). This places it within the expected range of cone photoreception. Indeed, a direct comparison revealed that the flash ERG of *Gnat1^−/−^* mice (in which cone photoreceptors remain functional) had a similar threshold, albeit significantly higher magnitude than that of TKO mice. However, we were unable to elicit a measurable response from TKO mice under light adapted conditions (Fig. 1C), indicating that other aspects of its photoresponse were atypical of cones. These two findings exclude one trivial explanation for the TKO ERG - that it reflects a simple genotyping error in our colony. Thus, as *Gnat1^+/+^* and *Gnat1^+/−^* mice retain scotopic light responses[2], while light adapted ERGs are recorded in *Cnga3^+/+^* and *Cnga3^+/−^* mice[3], the absence of these two ERG features confirms that the mice used in this experiment were homozygous knockout at both *Gnat1* and *Cnga3* loci.

**Figure 2:**
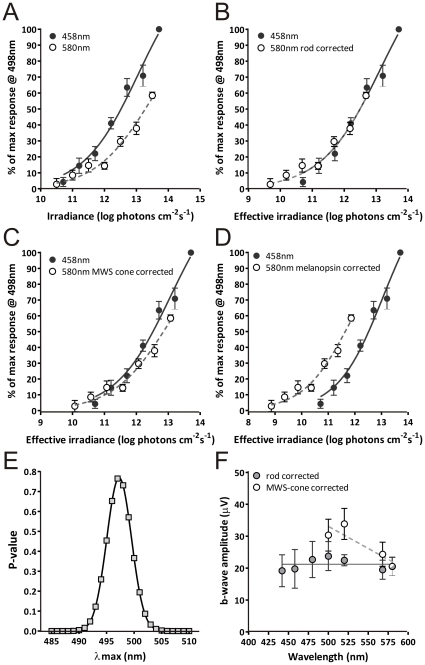
Spectral sensitivity of the TKO flash ERG. **A**, ERG b-wave amplitude (mean±SEM; n = 7) from TKO mice (expressed as % of maximum response at 458 nm) for 458 nm (blue) and 580 nm (yellow) flash stimuli. **B**–**D**, The same data plotted with stimulus irradiance at the two wavelengths normalized according to the spectral sensitivity of rod opsin (**B**), MWS opsin (**C**), and melanopsin (**D**). F test statistic allows the use of a single curve for the two wavelengths (p>0.05) when normalised for rod opsin, but not for MWS opsin (p<0.01), or melanopsin (p<0.0001). **E**. The relationship between p for the F statistic and the λ_max_ of the putative pigment used to normalize irradiance across the two wavelengths peaked close to 498 nm, the known spectral sensitivity of mouse rod opsin. **F.** b-wave amplitude (mean±SEM) for a range of monochromatic stimuli calculated to be isoluminant for rods (filled circles) or MWS-cones (open circles). Lines depict best fit by linear regression analysis, slope significantly different from 0 for the MWS-cone (p<0.05) but not rod (p>0.05) conditions. Note that the 580 nm datapoint contributes to both series.

To determine which photoreceptor drives the TKO ERG, we explored its spectral sensitivity. We initially constructed stimulus response relationships for monochromatic stimuli at 458 and 580 nm. The murine retina contains 4 known photopigments, UV and medium wavelength sensitive (MWS) cone opsins (peak sensitivity (λ_max_)  = 360 and 508 nm respectively); rod opsin (λ_max_  = 498 nm); and melanopsin (λ_max_ ≈480 nm). Although the TKO ERG was significantly less sensitive to 580 nm (Fig. 2A), the magnitude of this effect was far less than would be predicted if UV cone opsin were driving the response. In order to discriminate between the remaining 3 pigments, we normalized irradiances at the two wavelengths according to the known spectral efficiency function for each pigment (Fig. 2B–D). We then applied an F-test comparison to determine whether the normalized irradiance response functions could be fit with the same curve. We found that a single curve was appropriate for the two wavelengths when normalized for rod (p>0.05) but not either melanopsin (p<0.0001) or MWS cone opsins (p<0.01). Indeed, when we applied corrections based upon theoretical pigments with λ_max_ ranging from 484–511 nm we found that the two datasets could be fit by a single curve (F-test p>0.05) only when corrected according to the spectral sensitivity of pigments within a relatively narrow range (λ_max_  = 492–503 nm). A plot of the estimated probability for the two curves being fit by a single function against λ_max_ of the pigment used for normalization (Fig. 2E), produced a Gaussian distribution peaking at 497.3 nm, remarkably close to the mouse rod λ_max_ of 498 nm.

As a further test of the hypothesis that the TKO ERG is rod opsin dependent, we used the rod opsin spectral sensitivity function to calculate sub-saturating irradiances for monochromatic stimuli over the range 420 to 580 nm that were predicted to be rod-isoluminant. In agreement with this prediction we found that these stimuli evoked equivalent ERG responses over this wide wavelength range (Fig. 2F). By contrast, when stimuli in the range 500 to 580 nm were matched for MWS-cone sensitivity, we observed a negative correlation between b-wave amplitude and wavelength (Fig. 2G) implicating a shorter wavelength pigment as the origin of the response. Taken together, these spectral sensitivity analyses leave little doubt that the TKO ERG relies upon light absorption by rod opsin.

### TKO light responses survive U73122 application

**Figure 3:**
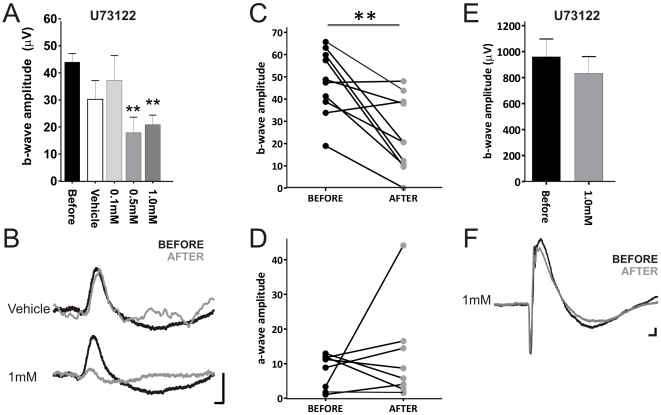
Impact of a PLC-β antagonist on the TKO ERG. Effects of the intravitreal injection of the PLC-β inhibitor U73122 on the b-wave amplitude (mean±SEM; **A**) and the average response waveform following vehicle or 1.0 mM U73122 in TKO mice (**B**). The same data plotted to show paired amplitudes of b-wave and a-wave before and after 1.0 mM U73122 for each individual (**C&D**). Effects of 1.0 mM U73122 intravitreal injection on wild type b-wave amplitude (**E**) and the average response waveform (**F**)**.** Sample size for TKO n = 4–6 for Vehicle, 0.1 mM and 0.5 mM U73122, and n = 10 for 1.0 mM U73122; for WT n = 4. Drug concentrations given in mM are for the injected preparation, final tissue concentration will be around 10× lower. Data in **A** analysed by one-way ANOVA and bonferroni post tests. Data in **C, D** and **E** analysed by paired two-tailed t-tests. * p<0.05; **p<0.p01. Scale bars in **B** and **F** = 50 ms (x-axis), 25 µV (y-axis).

The evidence for a Gnat1-independent rod ERG in TKO mice raises the question of what signaling cascade it employs. There have been suggestions that rods contain a phosphoinositide signaling pathway that may support light responses [13]. We tested this possibility using intravitreal injections of U73122, an antagonist for inosotyl phosphate phospholipase C (PLC) isoforms. We found a dose dependent decrease in b-wave amplitude with this drug (Fig. 3A–C). The simplest explanation for this result is that it reflects the predicted direct inhibition of the phototransduction cascade. However, other aspects of our results argue against this interpretation. Thus, if U73122 does inhibit phototransduction then the much smaller TKO a-wave should be abolished by this treatment. In fact, we observed no consistent effect on a-wave amplitude (Fig 3D).

**Figure 4:**
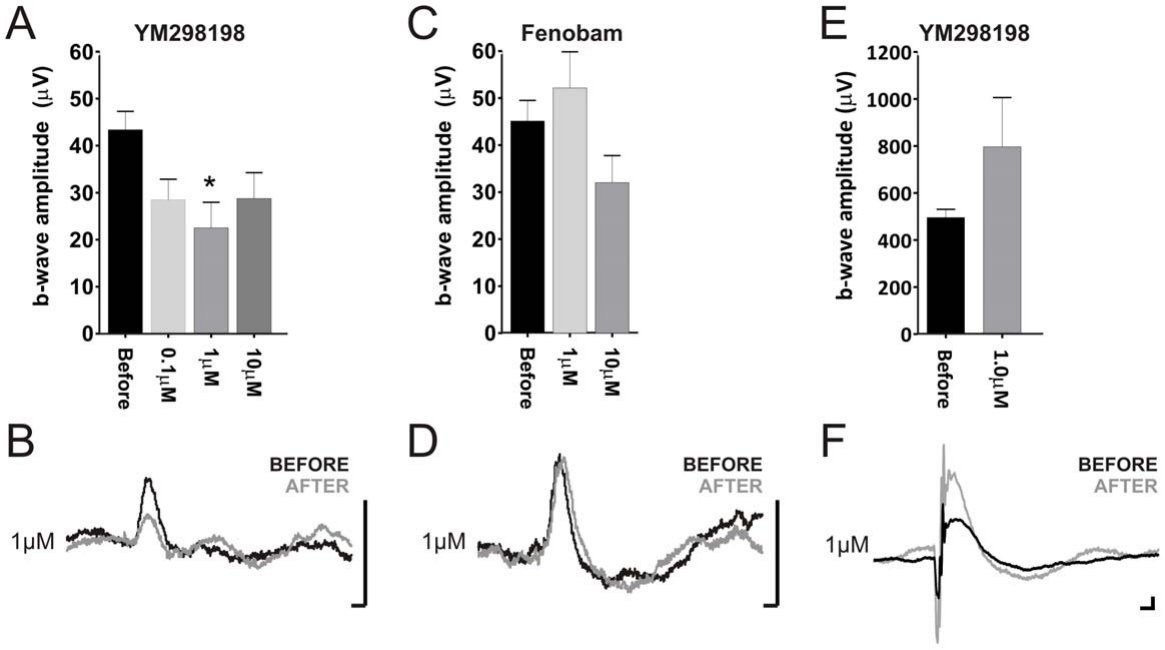
Pharmacology of the TKO ERG. Effects of intravitreal injection of the mGluR1 antagonist YM298198 (**A**, **B**, **E**, **F**) and the mGluR5 antagonist Fenobam (**C**, **D**) on b-wave amplitude (mean±SEM; **A**, **C**, **E**) and the average response waveform (**B**, **D**, **F**) in TKO (**A**&**B**, **C**&**D**) and wild type (**E**&**F**) mice. Sample for YM298198 n = 6 for TKO and 5 for WT; and for Fenobam n = 5 for TKO. All data show responses to a bright white flash (3.5 log_10_cd/m^2^). Drug concentrations given are for the injected preparation; final tissue concentration will be around 10× lower. Data analysed by one-way ANOVA and bonferroni post tests, except for **E** in which a paired t-test was employed. *<p0.05; **p<0.01. Scale bars in **B**, **D**, **F** = 50 ms (x-axis), 25 µV (y-axis).

An alternative explanation for the reduction in b-wave amplitude is that U73122 targets some aspect of the ON bipolar cell response. In support of this possibility we found that physiological concentrations of YM298198 (injected at 0.1–10 µM; estimated retinal concentration 10 nm–1 µM), an antagonist of the G_α_q PLC-coupled glutamate receptor mGluR1[14], reduced the TKO b-wave by a similar amount (Fig. 4A & B). Fenobam, which targets mGluR5[15], the other G_α_q-coupled glutamate receptor expressed in the retina[16], had no effect on the TKO ERG even at 10 µM (Fig. 4C & D). Neither U73122 nor YM298198 had a significant effect on the wild type ERG in our hands (Fig. 3 E & F; Fig. 4E & F) indicating that the processes giving rise to the TKO ERG are more sensitive to these agents than are those providing the majority of the wild type response. Taken together these results suggest that while mGluR1, and therefore PLC, influence the second order retinal response in TKO mice, the primary phototransduction mechanism in these animals is not PLC-dependent.

### A role for Gnat2 in the TKO ERG?

**Figure 5:**
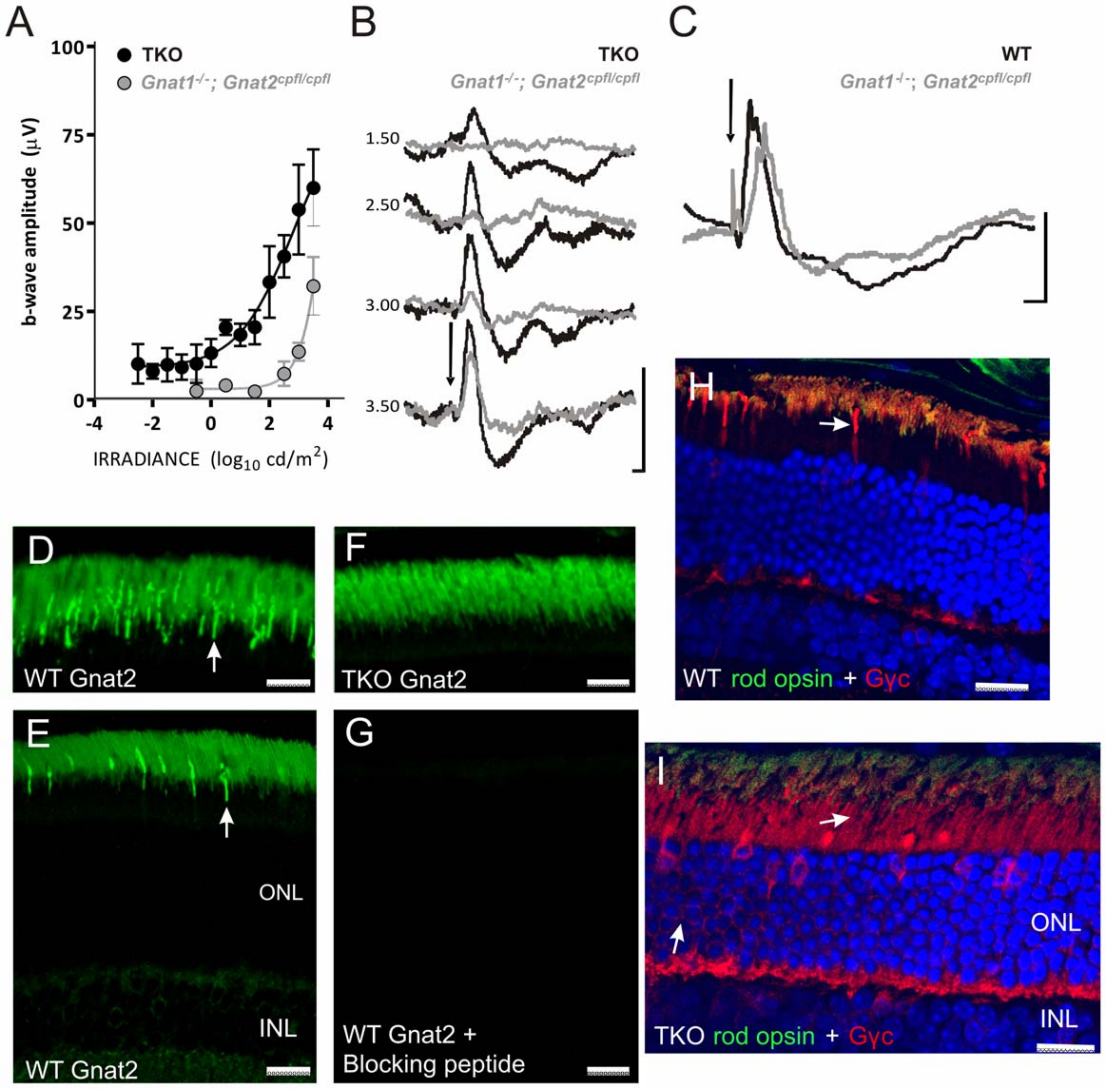
ERG responses in *Gnat1^−/−^;Gnat2^cpfl3/cpfl3^* mice. **A**, Comparison of b-wave amplitude of scotopic ERG in TKO and *Gnat1^−/−^;Gnat2^cpfl3/cpfl3^* mice (mean±SEM; n = 5 for each group). **B**, Representative scotopic ERG traces from TKO and *Gnat1^−/−^;Gnat2^cpfl3/cpfl3^* mice recorded in response to white flash stimuli (arrow depicts flash onset; scale bar =  50 ms (x-axis), 25 µV (y-axis); numbers to left are stimulus irradiance in log cd/m^2^). **C**, Representative traces for light-adapted ERGs in wildtype and *Gnat1^−/−^;Gnat2^cpfl3/cpfl3^* mice (arrow depicts flash onset; scale bar  = 50 ms (x-axis), 25 µV (y-axis)). Immunocytochemistry for Gnat2 using an anti-Gαt2 antibody in WT retina (**D-E**) revealed strong immunoreactivity in cones (arrows) with a lower level of staining found in rods. In TKO retina (**F**), staining in cones was absent, while immunoreactivity in rods persisted, even though these mice lack Gnat1, the most likely target of any cross-reactivity for this antibody. Pre-absorption with blocking peptide abolished staining in both rods and cones (**G**). Similarly, immunoreactivity for the γ subunit of cone transducin was detected in both cones and, to a lesser extent, rods of WT (**H**; arrow points to cones) and TKO (**I**; arrows point to rods) retinas. In WT retina there was a strong signal in cone outer segments, which was displaced to inner segments in TKO mice. Confocal settings were identical for images shown in D&F, E&G and H&I to facilitate comparison. Scale bars: **D**&**F** 10 µm, all others 20 µm.

Rod opsin can couple effectively to the cone α transducin (Gnat2)[17]. As the TKO ERG had functional characteristics similar to both rod and cone pathways and, as photoreceptors expressing elements of both rod and cone transduction cascades are reported in some species[18], [19], we wondered whether such a chimeric photoreceptor could form the origin of this light response. To test this possibility we crossed *Gnat1^−/−^* mice with animals carrying the *Gnat2^cpfl3^* allele, a missense mutation that results in a progressive loss of cone function[20]. A similar mouse model (*rd17*) has previously been reported to lack an ERG [17]. We used brighter stimuli and found that, in fact it was possible to elicit a measurable flash ERG from *Gnat1^−/−^;Gnat2^cpfl3/cpfl3^* mice. Nevertheless, both the magnitude and sensitivity of this response were substantially impaired compared to the TKO ERG (Fig. 5). The residual ERG of *Gnat1^−/−^;Gnat2^cpfl3/cpfl3^* mice probably originates in cones, as these animals retain a light adapted ERG (which is lost in TKO mice) and has been previously described in young *Gnat2^cpfl3/cpfl3^* animals. These data therefore indicate that the Gnat1-independent rod response is at least partially (and maybe completely) abolished by the *Gnat2^cpfl3^* mutation.

Although there is a secondary loss of rod function in *Gnat2^cpfl3/cpfl3^* mice, this effect is modest and evident only in animals >9 months of age [20]. It therefore seems unlikely that the loss of flash ERG responses at all but the highest stimulus intensities in the relatively young *Gnat1^−/−^;Gnat2^cpfl3/cpfl3^* mice studied here is a consequence of generalized rod dysfunction. Rather these data argue for a more specific involvement of Gnat2 in the Gnat1-independent rod response. In support of this possibility, we found immunocytochemical evidence for expression of both α and γ subunits of the cone transducin in wild type and TKO rods, albeit at low levels compared to cones (Fig. 5D–I).

### Activity in the thalamo-cortical visual pathway of TKO mice

**Figure 6:**
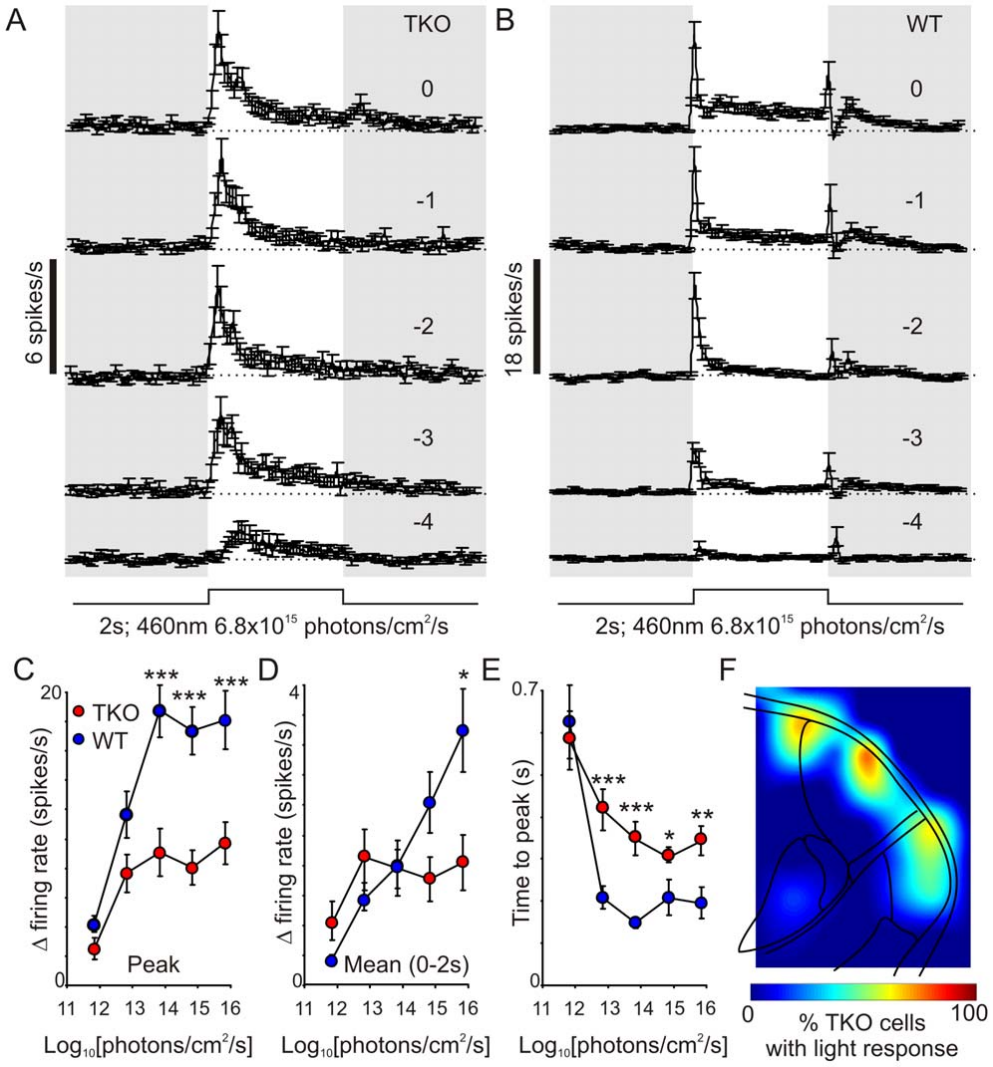
Visual responses in the lateral geniculate nucleus of TKO mice. (**A,B**) Average (±SEM) responses of TKO (**A**; n = 46) and wildtype (WT, **B**; n = 37) LGN neurons to 2s (460 nm) light pulses (note the difference in y-axis scaling between panels). Numbers above traces represent log_10_ [intensity], relative to the unattenuated intensity (6.8×10^15^ photons/cm^2^/s; 3.5 log cd/m^2^). (**C**–**D**) quantification of the responses of TKO and WT cells across each intensity showing (**C**) change in peak firing rate (from baseline), (**D**) change in mean firing rate across the 2s light stimulation and (**D**) time from light on to peak firing. Data were analysed by 2-way ANOVA followed by t-tests at each intensity; * = P<0.05, ** = P<0.01, *** = P<0.001. (**F**) projected anatomical localisation of light responsive TKO cells (based on 46 light responsive cells out of 253 total recorded from 4 mice), showing a relatively discrete distribution along the lateral margin of the LGN.

The presence of a measurable ERG in TKO mice raises the possibility that these animals may retain a wide array of light responses. To determine whether this could extend to aspects of pattern vision and perception we first searched for light evoked responses in the lateral geniculate nucleus using multitrode recording electrodes. We found that a 2s light step did indeed result in measurable light responses in this part of the brain (Fig. 6). 46 out of 253 identifiable single units (from 4 TKO mice) were visually responsive. All of these showed very similar profiles of light evoked activity. The highest sensitivity component was a transient increase in spike rate following light on. This became evident at 6.8×10^11^ photons/cm^2^/s (∼3 scotopic cd/m^2^, around the threshold for a measurable ERG response in these mice), and its magnitude increased, and latency decreased as a function of stimulus irradiance. Firing rate relaxed towards baseline following this transient peak, but remained elevated throughout light exposure especially at higher irradiances. At the highest intensity tested very weak responses to light off also appeared in some cells, although because of their magnitude these were hard to quantify accurately.

Light responses in TKO mice appeared in a restricted anatomical domain, along the lateral margin of the contralateral LGN, and were absent from more medial zones known to receive retinal input (Fig. 6F). This was not an artifact of our recording approach as we are able to record light evoked activity across the LGN in other mouse strains [21]. Analysis of the 37 light responsive single units identified in this wild type mouse were in accord with previous reports of light evoked activity in the mouse LGN [22], and our own recordings from other mouse strains [21], but quite different from those of TKO mice. Hence, light evoked spiking was of higher amplitude (Fig. 6C), reached maximal values more quickly (Fig. 6E) and at high light intensities tended to be more sustained (Fig. 6D) in this wild type than in any TKO mouse.

**Figure 7:**
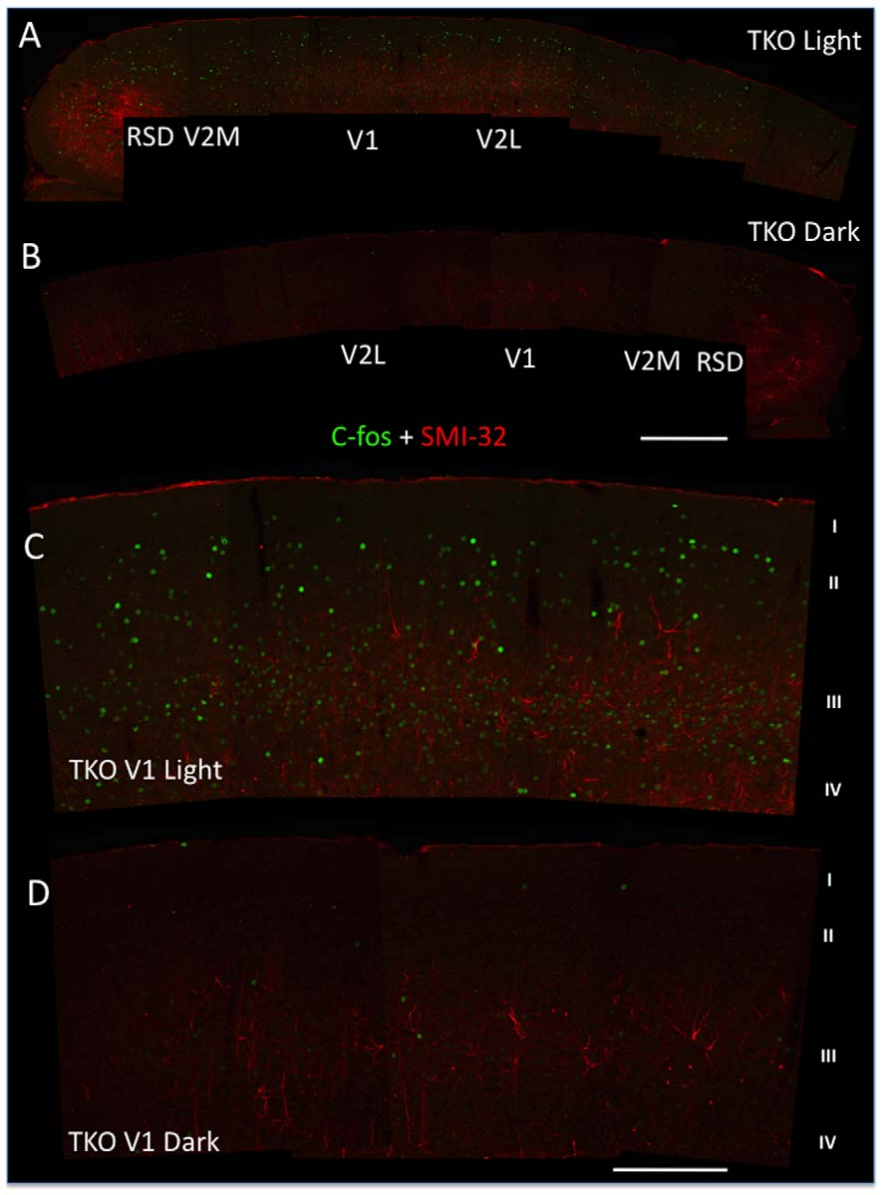
Light-driven neural activation in the visual cortex of TKO mice. Multiple immunostaining for c-fos (green) and SMI-32 (red) reveals a clear pattern of activation in response to light (**A** and **C**), relative to darkness (**B** and **D**). As shown at low magnification in **A**, light-driven c-fos induction was found in retrosplenial (RSD), primary (V1) and secondary (V2M/L) divisions of visual cortex. The V1 region from **A** and **B** is shown at higher magnification in **C** and **D** respectively. Light-driven neural activation, as visualized by c-fos positive nuclei, was seen throughout the different layers of primary visual cortex (I-IV). Scale bars: **A**–**B** 500 µm, **C**–**D** 200 µm.

A recent report that TKO mice exhibit behavioral light avoidance [23] indicates that visual information may be available to higher centers in this genotype. As a direct test of this possibility, we determined whether light exposure induced expression of c-fos in the cortex of TKO mice. Following exposure to a stimulus (90 minutes ∼1300 lux) shown previously to induce c-fos in wild type mice [10], we were able to identify c-fos immunoreactive nuclei in all layers of V1 together with neighbouring cortical zones V2 and RSD (as defined by SMI-32 immunoreactivity) in TKO animals (Fig. 7).

## Discussion

We show that despite lacking critical elements of all known phototransduction cascades, *Gnat1^−/−^;Cnga3^−/−^;Opn4^−/−^* mice retain a reproducible flash ERG and abundant physiological light responses in the thalamo-cortical visual pathway. Since ERGs are lost in *rd/rd cl* mice, a model of advanced outer retinal degeneration, these responses imply that either rods or cones retain residual phototransduction in TKO mice. As several stages of phototransduction precede light dependent closure of cyclic nucleotide gated channels [1], we might have expected that it would be the CNGA3 knockout whose impact on phototransduction would be incomplete. However, the spectral sensitivity of the TKO ERG implicates rod opsin as the origin of this response.

In the initial report of *Gnat1^−/−^* mice, Calvert et al. (2000) used suction electrode recordings to confirm that phototransduction was absent in rod photoreceptors. Those researchers did, however, detect light activated currents in a single photoreceptor (out of 213 tested). This cell responded to light with a spectral efficiency equivalent to that of rod opsin (albeit with slightly enhanced sensitivity at short wavelengths) but only at intensities within the cone sensitivity range. As these match the sensory characteristics of the TKO ERG it seems likely that the visual responses we report here reflect the activity of this rare rod response. If true, it follows that although Calvert *et al* (2000) detected only a single photoreceptor capable of Gnat1-independent phototransduction, such light sensitive cells are, in fact, sufficiently abundant to support visual responses.

The low sensitivity of the TKO ERG indicates that this residual rod opsin response cannot support conventional scotopic vision. In this regard, our data are consistent with the observation that human subjects with GNAT1 deficiency suffer CSNB [24]. However, the TKO response reveals that, in this context, loss of high sensitivity vision is not synonymous with a lack of rod photoreception. It follows that rods may remain a significant source of visual information in at least some people with CSNB.

An important area for future study will be the nature of the Gnat1-independent phototransduction cascade. The early receptor potential seems an unlikely origin. Firstly, by its nature the early receptor potential is a near instantaneous response to light exposure, whereas the TKO ERG displays relatively long response latencies. Secondly, the TKO ERG has relatively high sensitivity. Based on the method of Lyubarsky et al. [25] we estimate that a measurable ERG is recorded to a flash (15 ms, 1 cd/m^2^) inducing ∼8 photoisomerisations/rod. This implies signal amplification by a biochemical transduction cascade. In a previous study of *Gnat1^−/−^* mice, Woodruff et al. [26] reported that extremely bright flashes could elicit a slow current in all rods. They continued to provide evidence that it reflected closure of cGMP gated channels driven by light activated release of calcium. Such a transduction cascade has previously been suggested to function independent of Gnat2 in zebrafish cones[27]. In both of those cases very bright lights were used to evoke responses and, as we observe light responses even at fairly low intensities in TKO mice (around threshold for a cone-dependent ERG), the significance of those findings for our data (and for the rare rod response reported by Calvert et al. (2000)) is uncertain. It is certainly possible that there are two Gnat1-independent mechanisms, one of which is Ca-dependent and active in all rods at high light intensities; and another which is more sensitive and restricted to a subset of rods. Indeed, Woodruff et al [26] report diversity in the response of *Gnat1^−/−^* rods to bright flashes, with a small subset (∼5%) showing much stronger light activated currents than the rest.

We provide a preliminary test of two candidate cascades for the TKO ERG. There are many reports that rods contain elements of phosphoinositide signaling pathways, including G_α_11 and phospholipase C β4 ([13] and refs therein). Our data with U73122 provides at best partial support for the hypothesis that this is the origin of Gnat1-independent phototransduction. This PLC antagonist substantially inhibited the b-wave of the TKO ERG, but did not have a similar effect on the a-wave. This indicates that the primary phototransduction event in the TKO retina is not PLC dependent. The effects of the PLC antagonist on the b-wave would then have to originate with the second order response and we provide support for this possibility by showing that YM298198, an antagonist of the PLC-coupled mGluR1 receptor, has a similar effect on b-wave amplitude.

Our data provide more support for an involvement of Gnat2 in the TKO response. Thus, although *Gnat1^−/−^;Gnat2^cpfl3/cpfl3^* mice retain an ERG to a very high intensity flash, responses at moderate light levels (which are a feature of TKO mice) are lost. Could then rod opsin couple to Gnat2 in the TKO retina? Our immunocytochemical evidence for low-level Gnat2 expression in rods provides support for this possibility. This has not been reported previously by researchers using this, or other, antibodies against Gnat2[17], [20], [28], but given the low level staining we observe in rods compared with cones, others may have adjusted their protocols to eliminate what they assumed to be a non-specific signal and/or cross reactivity of the antibody with Gnat1. We exclude the possibility of cross reactivity with Gnat1 by studying Gnat1-less TKO rods, and have addressed the more general concern of non-specific hybridisation using a blocking peptide. The observation that mice carrying a cytotoxin (DTA) under control of the Gnat2 promoter display aggressive rod degeneration [29]provides indirect support for the concept of Gnat2 expression in rods, as this does not occur with other cone-specific promoters[30]. Nevertheless, if Gnat2 really is present in all rods this begs the question of why Calvert et al. (2000) found most *Gnat1^−/−^* rods to be largely insensitive to light. Furthermore, the association between Gnat2 and the Gnat1-independent rod response implied by our *Gnat1^−/−^;Gnat2^cpfl3/cpfl3^* data could, of course, be indirect.

Perhaps the most immediate practical implication of our findings comes from the new insight into the phenotype of *Gnat1^−/−^* mice that they provide. The *Gnat1^−/−^* mouse is a widely used model of rod-inactivation, partly because, unlike other lesions of rod phototransduction, it lacks significant secondary retinal degeneration. The appearance of rod opsin-driven visual responses in TKO mice indicates that, while these animals certainly lack conventional high sensitivity rod vision, it is not safe to assume that visual responses at higher light levels receive no rod input. This possibility should therefore been considered in experiments employing that genotype.

Along a similar line, the ERG responses we record in both *Gnat1^−/−^;Cnga3^−/−^;Opn4^−/−^* and *Gnat1^−/−^;Gnat2^cpfl3/cpfl3^* mice reveal that neither of the methods used to silence rod and cone phototransduction in these genotypes are completely successful. Consequently, they do not represent a superior alternative to current models employing retinal degeneration to generate rodless+coneless animals [12] to study melanopsin-based vision. In this regard, our findings also suggest an alternative potential interpretation for the recent report that *Gnat1^−/−^;Cnga3^−/−^* mice can use visual cues for maze navigation[31]. As these mice have heretofore been considered to rely solely on melanopsin for photosensitivity, this finding has been interpreted as evidence that melanopsin can support pattern vision in mice. Our data suggest an alternative possible explanation - that pattern vision in this model actually relies on the residual rod opsin photoresponse. Interestingly, Ecker and coworkers [31] report that TKO mice can use crude (light vs dark) visual cues to navigate a maze, but cannot distinguish gratings even at the lowest frequency tested (0.1cycles/°). Consequently, if the residual rod photoresponse were the origin of grating detection in their *Gnat1^−/−^;Cnga3^−/−^* mice, one would have to postulate a mechanism by which melanopsin could increase its acuity to the reported ∼0.15 cycles/°. This could take the form of melanopsin's known ability to drive pupil constriction[32], or more complex interactions between melanopsin photoreception and the activity of conventional visual pathways (e.g. [33]). Our data therefore call for further examination of the exciting possibility that melanopsin can contribute directly to pattern vision.
